# Effect of Artificial Intelligence Tutoring vs Expert Instruction on Learning Simulated Surgical Skills Among Medical Students: A Randomized Clinical Trial

**DOI:** 10.1001/jamanetworkopen.2021.49008

**Published:** 2022-02-22

**Authors:** Ali M. Fazlollahi, Mohamad Bakhaidar, Ahmad Alsayegh, Recai Yilmaz, Alexander Winkler-Schwartz, Nykan Mirchi, Ian Langleben, Nicole Ledwos, Abdulrahman J. Sabbagh, Khalid Bajunaid, Jason M. Harley, Rolando F. Del Maestro

**Affiliations:** 1 Neurosurgical Simulation and Artificial Intelligence Learning Centre, Department of Neurology and Neurosurgery, Montreal Neurological Institute and Hospital, McGill University, Montreal, Canada; 2 Faculty of Medicine and Health Sciences, McGill University, Montreal, Canada; 3 Division of Neurosurgery, Department of Surgery, College of Medicine, King Abdulaziz University, Jeddah, Saudi Arabia; 4 Clinical Skills and Simulation Center, King Abdulaziz University, Jeddah, Saudi Arabia; 5 Department of Surgery, College of Medicine, University of Jeddah, Jeddah, Saudi Arabia; 6 Department of Surgery, McGill University, Montreal, Canada; 7 Research Institute of the McGill University Health Centre, Montreal, Canada; 8 Institute for Health Sciences Education, McGill University, Montreal, Canada; 9 Steinberg Centre for Simulation and Interactive Learning, McGill University, Montreal, Canada

## Abstract

**Importance:**

To better understand the emerging role of artificial intelligence (AI) in surgical training, efficacy of AI tutoring systems, such as the Virtual Operative Assistant (VOA), must be tested and compared with conventional approaches.

**Objective:**

To determine how VOA and remote expert instruction compare in learners’ skill acquisition, affective, and cognitive outcomes during surgical simulation training.

**Design, Setting, and Participants:**

This instructor-blinded randomized clinical trial included medical students (undergraduate years 0-2) from 4 institutions in Canada during a single simulation training at McGill Neurosurgical Simulation and Artificial Intelligence Learning Centre, Montreal, Canada. Cross-sectional data were collected from January to April 2021. Analysis was conducted based on intention-to-treat. Data were analyzed from April to June 2021.

**Interventions:**

The interventions included 5 feedback sessions, 5 minutes each, during a single 75-minute training, including 5 practice sessions followed by 1 realistic virtual reality brain tumor resection. The 3 intervention arms included 2 treatment groups, AI audiovisual metric-based feedback (VOA group) and synchronous verbal scripted debriefing and instruction from a remote expert (instructor group), and a control group that received no feedback.

**Main Outcomes and Measures:**

The coprimary outcomes were change in procedural performance, quantified as Expertise Score by a validated assessment algorithm (Intelligent Continuous Expertise Monitoring System [ICEMS]; range, −1.00 to 1.00) for each practice resection, and learning and retention, measured from performance in realistic resections by ICEMS and blinded Objective Structured Assessment of Technical Skills (OSATS; range 1-7). Secondary outcomes included strength of emotions before, during, and after the intervention and cognitive load after intervention, measured in self-reports.

**Results:**

A total of 70 medical students (41 [59%] women and 29 [41%] men; mean [SD] age, 21.8 [2.3] years) from 4 institutions were randomized, including 23 students in the VOA group, 24 students in the instructor group, and 23 students in the control group. All participants were included in the final analysis. ICEMS assessed 350 practice resections, and ICEMS and OSATS evaluated 70 realistic resections. VOA significantly improved practice Expertise Scores by 0.66 (95% CI, 0.55 to 0.77) points compared with the instructor group and by 0.65 (95% CI, 0.54 to 0.77) points compared with the control group (*P* < .001). Realistic Expertise Scores were significantly higher for the VOA group compared with instructor (mean difference, 0.53 [95% CI, 0.40 to 0.67] points; *P* < .001) and control (mean difference. 0.49 [95% CI, 0.34 to 0.61] points; *P* < .001) groups. Mean global OSATS ratings were not statistically significant among the VOA (4.63 [95% CI, 4.06 to 5.20] points), instructor (4.40 [95% CI, 3.88-4.91] points), and control (3.86 [95% CI, 3.44 to 4.27] points) groups. However, on the OSATS subscores, VOA significantly enhanced the mean OSATS overall subscore compared with the control group (mean difference, 1.04 [95% CI, 0.13 to 1.96] points; *P* = .02), whereas expert instruction significantly improved OSATS subscores for instrument handling vs control (mean difference, 1.18 [95% CI, 0.22 to 2.14]; *P* = .01). No significant differences in cognitive load, positive activating, and negative emotions were found.

**Conclusions and Relevance:**

In this randomized clinical trial, VOA feedback demonstrated superior performance outcome and skill transfer, with equivalent OSATS ratings and cognitive and emotional responses compared with remote expert instruction, indicating advantages for its use in simulation training.

**Trial Registration:**

ClinicalTrials.gov Identifier: NCT04700384

## Key Points

**Question:**

How does feedback from an artificial intelligence (AI) tutoring system compare with training by remote expert instruction in learning a surgical procedure?

**Findings:**

In this randomized clinical trial including 70 medical students, learning a simulated operation achieved significantly higher performance scores when training with an AI tutor compared with expert instruction and a control with no feedback. Students’ cognitive and affective responses to learning with the AI tutor were similar to that fostered by human instructors.

**Meaning:**

These findings suggest that learning surgical skills in simulation was more effective with metric-based assessment and formative feedback on quantifiable criteria and actionable goals by an AI tutor than remote expert instruction.

## Teaser

This randomized clinical trial assesses artificial intelligence feedback, instructor feedback, and no feedback in simulated learning of surgical skills among medical students.

## Introduction

Mastery of bimanual psychomotor skills is a defining goal of surgical education,^1,2^ and wide variation in surgical skill among practitioners is associated with adverse intraoperative and postoperative patient outcomes.^3,4^ Novel technologies, such as surgical simulators using artificial intelligence (AI) assessment systems, are improving our understanding of the composites of surgical expertise and have the potential to reduce skill heterogeneity by complementing competency-based curriculum training.^5,6,7^ Virtual reality simulation and machine learning algorithms can objectively quantify performance and improve the precision and granularity of bimanual technical skills classification.^8,9,10^ These systems may enhance surgical educators’ ability to develop more quantitative formative and summative assessment tools to manage future challenging pedagogic requirements. The COVID-19 pandemic has significantly altered surgical trainees’ ability to obtain intraoperative instruction necessary for skill acquisition,^11^ and innovative solutions, such as AI-powered tutoring systems, may help in addressing such disruptions.^12^

An AI tutoring system refers to an educational platform driven by computer algorithms that integrate assessment with personalized feedback.^13^ Our group has developed an AI tutoring system called the *Virtual Operative Assistant* (VOA) that uses a machine learning algorithm, support vector machine, to classify learner performance and provide goal-oriented, metric-based audiovisual feedback in virtual reality simulations.^14^ Following the competency-based medical education model of the Royal College of Physicians and Surgeons of Canada,^15^ and to mitigate extrinsic cognitive load through segmentation,^16^ the system guides learners in 2 steps: first, helping trainees reach competency in safety metrics and second, evaluating metrics associated with instrument movement and efficiency.^14^ The VOA AI tutoring system is designed for surgical simulation training, but its effectiveness compared with conventional surgical instruction is unknown.

Expert-led telementoring and virtual clerkships use technologies, such as augmented reality headsets and videotelephony software, for supervision and feedback.^17,18^ With the ongoing pandemic, these adaptations may provide alternatives to intraoperative surgical instruction.^19^ For this study, we followed the criterion standards of assessment and debriefing in surgical education, Objective Structured Assessment of Technical Skills (OSATS)^20^ and Promoting Excellence and Reflective Learning in Simulation (PEARLS) debriefing guide,^21^ to design a standardized expert-led remote training as the traditional control.

We sought to investigate VOA’s educational value by comparing it with remote expert instruction in enhancing technical performance and learning outcomes of medical students during brain tumor resection simulations and eliciting emotional and cognitive responses that are associated with supporting learning. Our hypothesis was that VOA feedback would be similar to remote expert instruction in performance outcomes but lead to stronger negative emotions and higher cognitive load.

## Methods

This multi-institutional instructor-blinded randomized clinical trial was approved by McGill University Health Centre Research Ethics Board, Neurosciences–Psychiatry. All participants signed an informed consent form prior to participation. This report follows the Consolidated Standards of Reporting Trials involving AI (CONSORT-AI)^22^ and Best Practices for Machine Learning to Assess Surgical Expertise.^23^ The trial protocol and statistical analysis plan are available in Supplement 1.

### Participants

Medical students with no surgical experience were invited to voluntarily participate. Recruitment information was shared among student networks, social media, and interest groups. Selection was based on meeting inclusion criterion: enrollment in Medicine Preparatory or first or second year of a medical program in Canada. Our exclusion criteria were participation in surgical clerkship or previous experience with the virtual reality simulator used in this study (NeuroVR; CAE Healthcare).

### Randomization

**Figure 1.**
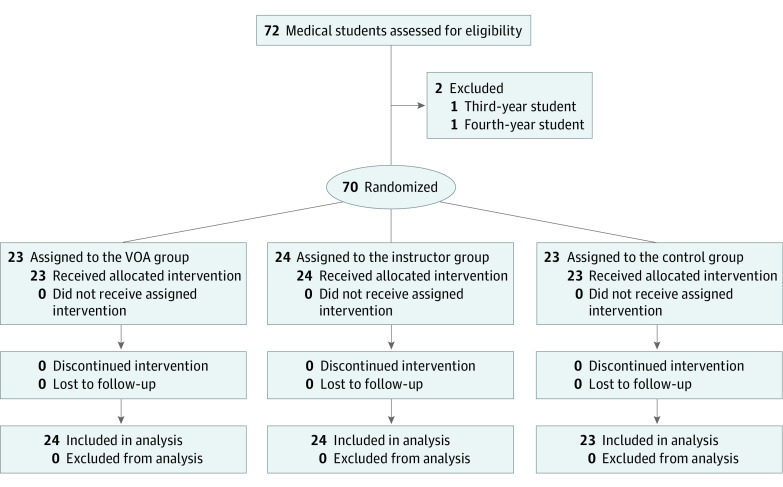
Participant Recruitment Flowchart

Students were stratified by sex and block randomized to 3 intervention arms, allocation ratio of 1:1:1, using an internet-based, computer-generated random sequence.^24^ Group allocation was concealed by the study coordinator, and instructors were notified of appointment times 1 day in advance for scheduling purposes. The participant recruitment flowchart is outlined in Figure 1.

### Study Procedure

After participants provided written consent, they completed a background information questionnaire that recorded baseline emotions using the Medical Emotion Scale (MES),^25^ experiences that may influence bimanual dexterity (ie, video games,^26^ musical instruments^27^), deliberate practice (ie, competitive sports^28^), or prior virtual reality navigation. Students were not informed of the trial purpose or assessment metrics. Participants performed 5 practice simulated tumor resections^29^ (eFigure 1 in Supplement 2), followed by feedback (intervention) or no feedback (control), then completed 1 realistic tumor resection simulation^30^ (eFigure 2 in Supplement 2) to evaluate learning and transfer of technical skills. The MES self-report was administered again on completion of the fifth and sixth resections to assess participants’ emotions during and after the learning session, respectively, and the Cognitive Load Index^31^ self-report was used to measure cognitive load after training.

### Simulator

The tumor resection simulator, NeuroVR, simulates neurosurgical procedures on a high-fidelity platform that recreates the visual, auditory, and haptic experience of resecting human brain tumors (eFigure 3 in Supplement 2).^32^ Because this simulator records timeseries data of users’ interaction in the virtual space,^33^ machine learning algorithms have been demonstrated to successfully differentiate surgical expertise based on validated performance metrics.^8,9,10,34^

### Virtual Reality Tumor Resection Procedures

Subpial resection is a neurosurgical technique in oncologic and epilepsy surgery that requires coordinated bimanual psychomotor ability to resect pathologic tissue with preservation of surrounding brain and vessels.^35^ The student’s objective was to remove a simulated cortical tumor with minimal bleeding and damage to surrounding tissues using a simulated aspirator in the dominant hand and a simulated bipolar forceps in the nondominant hand (Video).^29,30^ Participants received standardized verbal and written instructions on instrument use and performed orientation modules to understand each instrument’s functions. Individuals had 5 minutes to complete each practice resection and 13 minutes for the realistic resection. The first practice subpial resection was considered baseline performance.

**Video.** Virtual Reality Brain Tumor Resection on the NeuroVR Partial recording from a single participant performing the practice and the realistic virtual reality subpial resection task. The bipolar is held with the nondominant hand and appears on the left. The aspirator is held in the dominant hand and appears on the right.

### Interventions

Participants were allocated 5 minutes between each resection session to receive the intended intervention. Both experimental arms followed principles of deliberate practice guided by self-regulated learning,^36,37^ in which formative assessment enables finding areas of growth, setting goals, and adopting strategies that enhance competence.^38^ The feedback received and progress toward learning objectives were monitored by either the VOA or an instructor.

#### VOA AI Tutoring

VOA estimates a competence percentage score and a binary expertise classification based on 4 metrics: assessment criteria selected through expert consultation and statistical, forward, and backward support vector machine feature selection.^14^ Competence is evaluated in 2 steps, safety and instrument movement, each associated with 2 metrics: mean bleeding rate and maximum bipolar force application for step 1, and mean instrument tip separation distance and mean bipolar acceleration for step 2. Learners must achieve expert classification for safety metrics in step 1 before moving to step 2 to learn instrument movement metrics and achieve competency. Individuals classified as novice in any metric receive automated audiovisual feedback (eFigure 4 in Supplement 2).^14^

#### Remote Expert Instruction

Delivering traditional apprenticeship learning during the COVID-19 pandemic for a controlled experiment requires steps that minimize contact and ensure consistency. Two senior neurosurgery residents (M.B. and A.A., postgraduate year 5) who had experience performing human subpial resection procedures completed standardized training (eAppendix 1 in Supplement 2) to perform simulations within consultants’ benchmarks, reliably rate on-screen performances using a modified OSATS visual rating scale,^39^ and provide feedback from a modified PEARLS debriefing script.^21^ Instructors were blinded to AI assessment metrics. Prior to recruitment, the OSATS scale demonstrated good internal consistency (α = 0.82 [95% CI, 0.77 to 0.87]) and instructors achieved good interrater reliability (intraclass correlation coefficient, 0.84 [95% CI, 0.79 to 0.88]).

Each participant’s live on-screen practice performance was assessed remotely by 1 randomly selected instructor (eFigure 5 in Supplement 2), who completed an assessment sheet (eAppendix 2 in Supplement 2). During debriefing, instructors followed a modified PEARLS script and provided feedback from a list of instructions, suggested by consultants, depending on students’ competency. The eTable in Supplement 2 contains details on feedback interventions.

#### Control Group

Control participants received no performance assessment or feedback and were instructed to use the time between simulations to reflect and set goals for the following trial. This follows principles of experiential learning through active experimentation and reflective observation,^40^ establishing a baseline for performance improvement and learning with no feedback.

### Outcome Measures

The coprimary outcome was the interaction effect of feedback on surgical performance improvement over time during 5 practice resections, measured by the Intelligent Continuous Expertise Monitoring System (ICEMS) Expertise Score: the mean of expertise predictions (range, −1.00 to 1.00, reflecting novice to expert rating) computed for every 0.2-second of the procedure, by a deep learning algorithm using a long short-term memory network with 16 input performance metrics from simulator’s raw data.^34^ The second coprimary outcome was learning and skill retention, evaluated based on realistic tumor resection performance by both the ICEMS and blinded OSATS assessment. The OSATS rubric contains 6 performance categories, each rated on a 7-point Likert scale (eAppendix 2 in Supplement 2). Secondary outcomes were differences in the strength of emotions before, during, and after training and cognitive demands required by each intervention. These were measured by self-report on the MES for emotional strength^25^ and the Cognitive Load Index for cognitive demands^31^ on a 5-point Likert scale.

### Statistical Analysis

Ad hoc analysis to achieve 80% statistical power (β = 0.20), estimating moderate primary outcome effect of 35%, with 2-sided test at α = .05, revealed a minimum of 23 participants were required for each intervention arm. Collected data were examined for outliers and normality. Levene test for equality of variance and Mauchly test of sphericity met assumptions of analysis of variance (ANOVA). Two-way mixed ANOVA investigated the interaction of group assignment (between-participants) and time (within-participant) on learning curves and emotion self-reports. One-way ANOVA tested between-group differences in learning, cognitive load, and OSATS scores. Baseline performance was assigned as a covariate in the mixed model. Repeated measures ANOVA examined within-participant changes of performance in each group. Significance was set at *P* < .05. *P* values were adjusted by Bonferroni correction for multiple tests. All statistical analyses were performed on SPSS statistical software version 27 (IBM). Expertise Score predictions were conducted in MATLAB release 2020a (MathWorks). Data were analyzed from April to June 2021.

## Results

**Figure 2.**
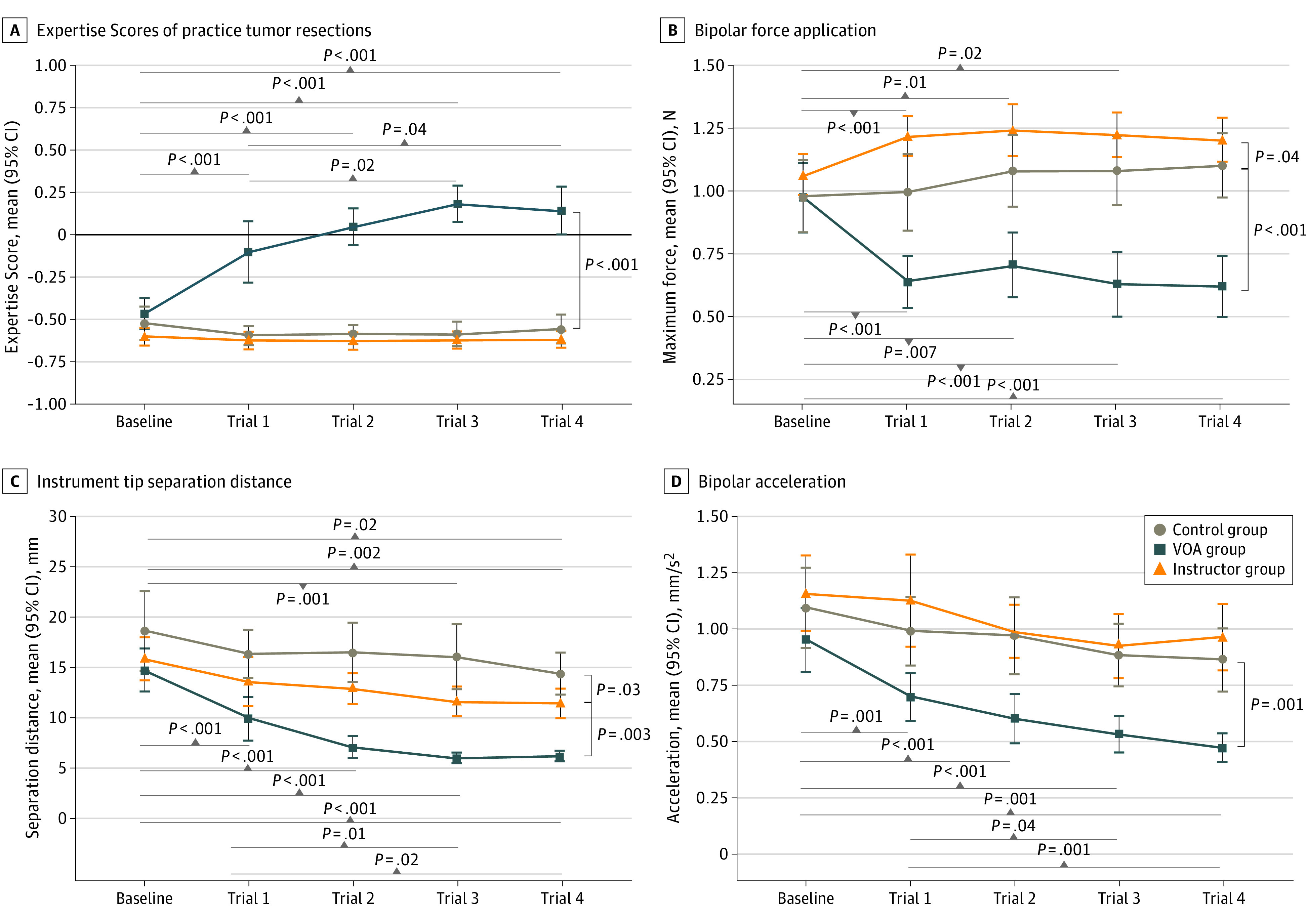
Performance Assessment in the Practice Tumor Resections A, Negative scores indicate a novice; and a positive score, a more expert performance. Scores in each trial are the mean of all estimations made for every 200 milliseconds of the simulated procedure (approximately 1500 predictions for a 5-minute practice scenario). B, Maximum bipolar force application is a recording of the highest amount of force applied with the bipolar during the entire operation. C, Mean instrument tip separation distance measured as the mean distance between the aspirator and the bipolar tips. D, Mean bipolar acceleration measured as the rate of change in the bipolar instrument’s velocity. Error bars indicate 95% CIs; and VOA, Virtual Operative Assistant.

A total of 70 medical students (41 [59%] women and 29 [41%] men; mean [SD] age, 21.8 [2.3] years) from 4 institutions (McGill University, 32 students [46%]; Laval University, 19 students [27%]; University of Montreal, 17 students [24%]; University of Sherbrooke, 2 students [3%]) were randomized, including 23 students in the VOA group, 24 students in the instructor group, and 23 students in the control group. Distribution of baseline characteristics was balanced among groups (Table). All included participants completed the training, and no one was lost to follow-up. A total of 350 practice resections and 70 realistic resections were scored by the ICEMS. Blinded experts evaluated 70 video recordings of realistic performances using the OSATS scale. There were no statistically significant differences among groups in baseline performance (Figure 2A). At baseline, mean Expertise Scores were −0.57 (95% CI, −0.66 to −0.48) points in the VOA group, −0.60 (95% CI, −0.66 to −0.55) points in the instructor group, and −0.53 (95% CI, −0.62 to −0.43) points in the control groups. All VOA group participants passed the safety module (step 1) and 14 students (61%) completed instrument movements competency (step 2) by the end of training (eFigure 6 in Supplement 2).

**Table.** Demographic Characteristics of Included Participants

Characteristic	Medical students, No. (%)		
	Control group (n = 23)	VOA group (n = 23)	Instructor group (n = 24)
Age, mean (SD), y	21.7 (2.4)	21.9 (2.5)	21.8 (2.1)
Sex			
Men	9 (39)	10 (43)	10 (42)
Women	14 (61)	13 (57)	14 (58)
Undergraduate medical training level			
Medicine Preparatory^a^	9 (39)	10 (43)	7 (29)
First year	8 (35)	8 (35)	9 (38)
Second year	6 (26)	5 (22)	8 (33)
Institution			
McGill University	14 (61)	8 (35)	10 (42)
University of Montreal	3 (13)	7 (30)	7 (29)
University of Laval	6 (26)	7 (30)	6 (25)
University of Sherbrooke	0	1 (5)	1 (4)
Dominant hand			
Right	23 (100)	21 (91)	22 (92)
Left	0	2 (9)	2 (8)
Interest in pursuing surgery, mean (SD)^b^	3.7 (1.0)	3.9 (1.1)	3.8 (1.2)
Play video games, h/wk			
Not at all	15 (65)	15 (65)	16 (67)
1-5	5 (22)	6 (26)	5 (21)
6-10	2 (9)	2 (9)	2 (8)
>11	1 (4)	0	1 (4)
Play musical instruments			
Yes	12 (52)	8 (35)	13 (54)
No	11 (48)	15 (65)	11 (46)
Did competitive sports in the past 5 y			
Yes	12 (52)	17 (74)	17 (71)
No	11 (48)	6 (26)	7 (29)
Prior VR experience in any domain			
None	14 (61)	12 (52)	12 (50)
Passive (eg, videos)	8 (35)	10 (43)	9 (38)
Active (eg, games, simulation)	1 (4)	1 (5)	3 (12)
Prior experience with any VR surgical simulator			
Yes	1 (4)	0	0
No	22 (96)	23 (100)	24 (100)

Abbreviations: VOA, Virtual Operative Assistant; VR, virtual reality.

^a^ Medicine Preparatory is a 1-year preparatory program for graduates of the Quebec Collegial system who have been offered a position from the medical program of McGill University or University of Montreal.

^b^ Rated on a Likert Scale (1-5), with 1 indicating less interest and 5 indicating more interest.

### Performance During Practice Tumor Subpial Resection

At completion, the mean Expertise Scores were 0.14 (95% CI, 0.01 to 0.28) points in the VOA group, −0.62 (95% CI, −0.68 to −0.57) points in the instructor group, and −0.56 (95% CI, −0.65 to −0.47) points in the control group. Mixed ANOVA demonstrated that within-participant performance changes depended on the type of feedback, with the VOA feedback group achieving a difference of 0.66 (95% CI, 0.55 to 0.77) points higher compared with the instructor group (*P* < .001) and 0.65 (95% CI, 0.54 to 0.77) points higher compared with the control group (*P* < .001) (Figure 2A). Mean Expertise Scores in instructor and control groups were not significantly different.

The VOA group demonstrated Expertise Scores improvements between trials (Figure 2A). Pairwise comparisons demonstrated that learners performed significantly better than baseline after AI tutoring feedback (mean difference vs baseline: trial 1, 0.37 [95% CI, 0.18 to 0.56] points; *P* < .001; trial 2, 0.51 [95% CI, 0.29 to 0.74] points; *P* < .001; trial 3, 0.65 [95% CI, 0.41 to 0.89] points; *P* < .001; trial 4, 0.61 [95% CI, 0.36 to 0.86] points; *P* < .001). There was significant improvement from trial 1 to trial 3 (mean difference, 0.28 [95% CI, 0.55 to 0.02] points; *P* = .02) and trial 1 to trial 4 (mean difference, 0.24 [95% CI, 0.00 to 0.49] points; *P* = .04). Learning curves demonstrate steady improvement from baseline to trial 3 that plateaued at trials 3 and 4. Three VOA feedback instances resulted in mean group performance higher than 0.00 points, the ICEMS novice threshold (Figure 2A).

Of the 4 VOA metrics used for competency training, 3 demonstrated improvement in VOA group and significant differences compared with the instructor and control groups (maximum bipolar force application, mean instrument tip separation distance, and mean bipolar acceleration) (Figure 2B-D). There was no significant difference among groups in bleeding rate owing to wide participant variability in this metric. VOA feedback was more effective in enhancing metric scores compared with expert instruction, and compared with control, remote expert feedback significantly reduced mean instrument tip separation distance (mean difference, –3.28 [95% CI, –6.36 to –0.21] mm; *P* = .03) (Figure 2C). Of the 16 ICEMS metrics not trained by the VOA, 8 significantly improved in the VOA group compared with instructor and control groups, suggesting that feedback on 4 AI-selected safety and instrument movement metrics resulted in improved bimanual psychomotor performance in other benchmark metrics.

### Realistic Tumor Resection Performance

**Figure 3.**
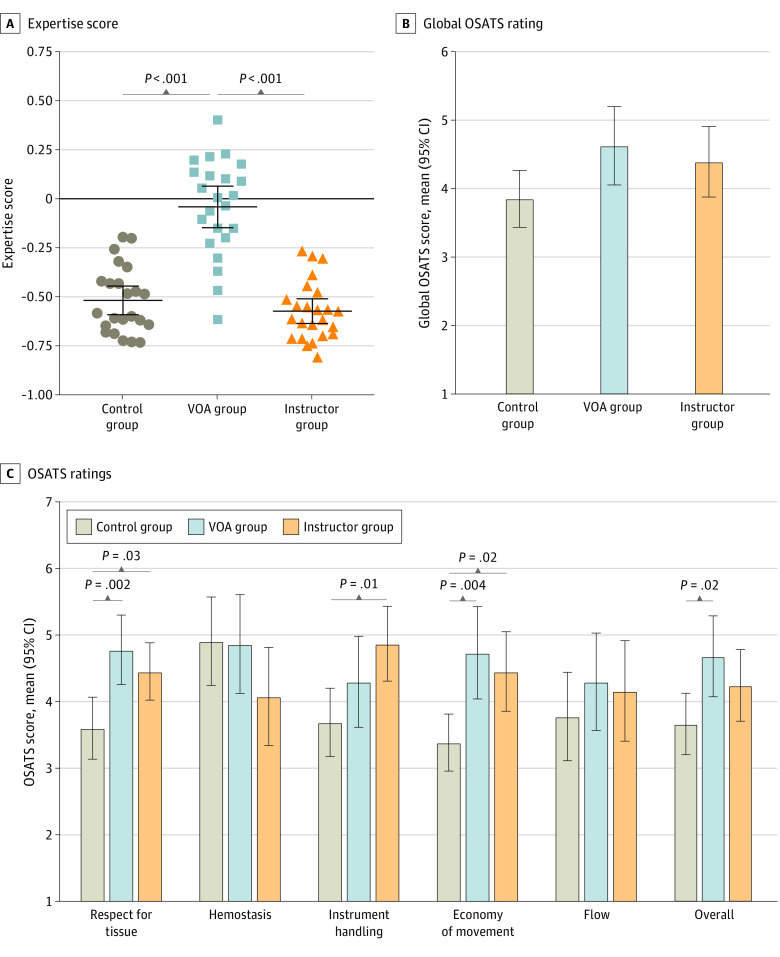
Performance Assessment in the Realistic Tumor Resection Error bars indicate 95% CIs; OSATS, Objective Structured Assessment of Technical Skills; and VOA, Virtual Operative Assistant.

The VOA group achieved significantly higher Expertise Scores in the realistic subpial resection than instructor (mean difference, 0.53 [95% CI, 0.40 to 0.67] points) and control (mean difference, 0.49 [95% CI, 0.34 to 0.61] points; *P* < .001) groups (Figure 3A). Global OSATS ratings of realistic subpial resections showed no significant difference between the VOA group (mean score, 4.63 [95% CI, 4.06 to 5.20] points) and the instructor group (mean score, 4.40 [95% CI, 3.88 to 4.91] points; mean difference, 0.23 [95% CI, −0.59 to 1.06] points; *P* = .78) or the control group (3.86 [95% CI, 3.44 to 4.27] points; mean difference, 0.78 [95% CI, −0.06 to 1.61] points; *P* = .07), consistent with an equivalent qualitative performance outcome. In OSATS subscores and compared with the control group, feedback significantly improved participants’ respect for tissue (mean difference: VOA, 1.17 [95% CI, 0.40 to 1.95] points; *P* = .002; instructor, 0.85 [95% CI, 0.08 to 1.62] points; *P* = .03) and economy of movement (mean difference: VOA, 1.35 [95% CI, 0.39 to 2.31] points; *P* = .004; instructor, 1.07 [95% CI, 0.12 to 2.02] points; *P* = .02). Compared with the control group, expert instruction significantly enhanced instrument handling (mean difference, 1.18 [95% CI, 0.22 to 2.14] points; *P* = .01) and VOA resulted in significantly higher OSATS overall subscore (mean difference, 1.04 [95% CI, 0.13 to 1.96] points; *P* = .02) (Figure 3C). Completing VOA’s instrument movement competency correlated significantly with higher economy of movement (Pearson *r* = 0.25, *P* = .03), suggesting successful acquisition of the relevant competency.

### Emotions and Cognitive Load

**Figure 4.**
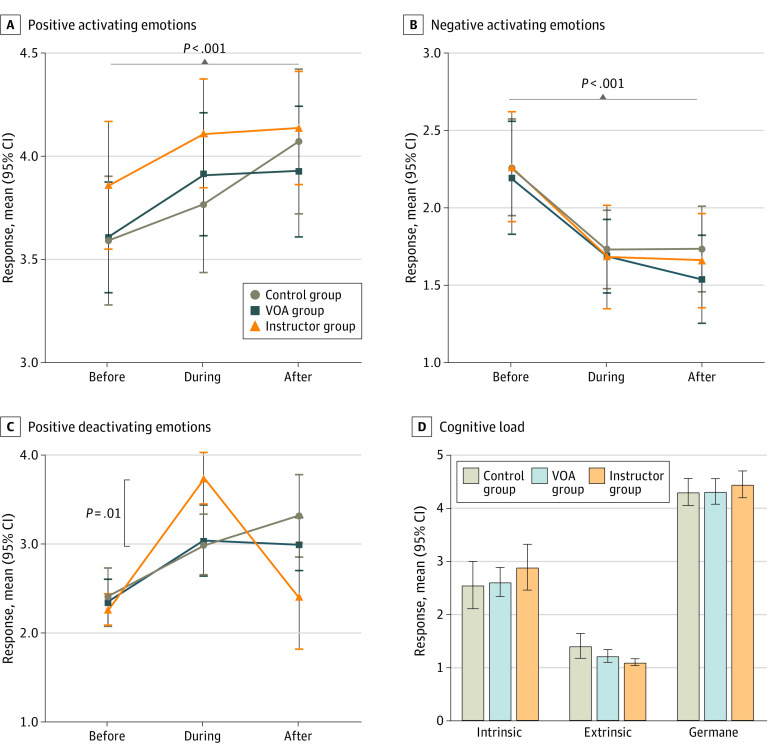
Emotions and Cognitive Load Throughout the Simulation Training Positive activating emotions include happy, hopeful, grateful (A), and negative activating emotions include confusion and anxiety (B). Error bars indicate 95% CIs; and VOA, Virtual Operative Assistant.

In within-participant analysis, there was a significant increase in positive activating emotions (after vs before mean difference, 0.36 [95% CI, 0.16 to 0.55] points; *P* < .001) and a significant decline in negative activating emotions (after vs before mean difference, –0.59 [95% CI, –0.85 to –0.34] points; *P* < .001) throughout the simulation training. The significant interaction effect in positive deactivating emotions demonstrated that instructor group participants felt more relieved and relaxed during training compared with learners in VOA (mean difference, 0.75 [95% CI, 0.19 to 1.31] points; *P* = .006) and control groups (mean difference, 0.71 [95% CI, 0.14 to 1.27] points; *P* = .01) (Figure 4A-C). No between-participant difference in intrinsic, extrinsic, and germane cognitive load were found (Figure 4D).

### AI Intervention Acceptance

To assess student acceptance of the AI intervention, we administered a poststudy questionnaire to all 23 students of the VOA group, and 22 students (96%) reported that they would prefer to learn from both expert instruction and AI tutoring. Additionally, only 1 student (4%) reported they preferred AI tutoring only, and no student reported they preferred expert instruction only.

## Discussion

This randomized clinical trial is the first study, to our knowledge, that compares the effectiveness of an AI-powered tutoring system with expert instruction in surgical simulation while assessing affective and cognitive response to such instruction. Surgical performance is an independent factor associated with postoperative patient outcomes,^41^ and technical skills acquired in simulation training improve operating room performance.^42,43,44^ Repetitive practice in a controlled environment and educational feedback are key features of simulation-based surgical education^45^; however, use of autonomous pedagogical tools in simulation training is limited.

In this randomized clinical trial, our findings demonstrated effective use of AI tutoring in surgical simulation training. VOA feedback improved performance during the practice and realistic simulation scenarios, measured quantitatively by Expertise Scores, and enhanced operative quality and students’ skill transfer, observed by OSATS during the realistic tumor resection. Objective metric-based formative feedback through AI tutoring demonstrated advantages compared with remote expert instruction. It helped students achieve higher expertise by bringing awareness to their metric goals during resections and setting measurable performance objectives, 2 effective strategies of learning theory.^46^ Feedback on AI-selected metrics had an extended effect on supplementary performance criteria used in both OSATS and ICEMS. VOA’s learning platform is flexible and allows learners with different levels of expertise to practice and receive personalized formative feedback based on interest and time availability. This AI intervention saved approximately 53 hours of expert supervision and formative assessment over 13 weeks compared with the instructor group while resulting in comparable OSATS scores. VOA did not bring participants’ Expertise Scores to the level of senior experts (ie, ICEMS >0.33),^34^ suggesting areas for future research and improvement. More research is needed to understand which surgical procedures lend themselves best to AI interventions, but this study provides evidence that this brain tumor resection technique may be an appropriate candidate.

In contrast to our hypothesis and previous reports, in which learning with an AI tutor elicited negative emotions, impairing students’ use of self-regulated learning strategies,^47,48^ learning bimanual tumor resection skills with VOA demonstrated a gradual decline in negative activating emotions with an overall increase in positive emotions, similar to human instruction. Encouragingly, VOA participants did not report this learning experience required significantly higher cognitive demands compared with the other interventions, demonstrating clear and comprehensible AI tutoring feedback that required minimal extraneous load.

Although the full impact of COVID-19 on surgical education remains unclear,^49^ it is important to prepare for future challenges through focused research and further development of effective remote learning platforms.^50^ We report 2 potential methods to address remote learning, both with demonstrated ability to enhance task performance better than control. Comparing efficacy between the 2 interventions arms of this trial is up to interpretation and limited to the primary outcome measures used. Curriculum coherence is a fundamental principle in education that is achieved in part by the alignment of intended learning outcomes and instructional activities with the assessment criteria.^51,52^ Following this principle in randomized trials involving educational interventions may create a potential overlap between the primary outcome measures and the pedagogical tools used during training. In this study, 4 of 16 ICEMS metrics were learning objectives of the VOA and all 6 OSATS categories were learning objectives of the instructor group; therefore, the use of either tool alone as a primary outcome may lead to bias toward better performance for one group. The VOA’s more flexible and time-efficient approach, in addition to its similar OSATS outcome and its extended effect on ICEMS’s remaining performance metrics, demonstrated that AI tutoring may have some advantages compared with remote expert instruction.

Consistent with previous studies,^53,54^ our findings suggest that scripted feedback by instructors established a supportive learning environment where participants felt stronger positive deactivating emotions during practice; however, this did not result in greater performance. Studies suggest that there is no statistically significant difference in complication rates, operative time, and surgical outcomes between telementoring and in-person instruction,^55,56^ but there is limited evidence comparing their educational effectiveness on technical performance. In this study, remote instruction was inferior to AI tutoring based on quantifiable metrics, but further research is necessary to determine if that remains the case with in-person coaching. Our remote-based method was considered feasible by instructors because they could easily join to provide virtual debriefing and technical instruction.

The AI algorithm used in this study failed to detect performance improvements in the instructor group according to OSATS ratings for practice and realistic scenarios (eFigure 7 in Supplement 2). OSATS categories, like instrument handling, describe a subjective qualitative composite of actions that AI systems have difficulty measuring from raw data. ICEMS functions at a deeper level by analyzing the interaction of several underlying metrics that contribute to expertise. These systems may be less able to assess operative strategies, such as a systematically organized tumor resection plan, that students may acquire more readily from expert instruction. These types of procedural instruction may take more educator time to become apparent as changes in learners’ metrics scores. Our findings suggest that monitoring specific AI-derived expert performance metrics, such as bipolar instrument’s acceleration and providing personalized quantitative learner feedback on these metrics, is an efficient method to guide behavioral changes toward a higher operative quality. However, integrating metric objectives with the task goals may be challenging and may require expert input. Most participants (96%) reported that they would prefer learning with feedback from both expert instruction and AI tutoring, suggesting complementary features from both methods could enhance the learning experience. With increasing efforts to capture live operative data,^57^ combining intraoperative use of AI tutoring and expert surgical instruction may accelerate the path to mastery.

### Limitations

This study has some limitations. Although the AI-powered virtual reality simulation platform used in this study allows detailed quantitative assessment of bimanual technical skills, it fails to capture the full spectrum of competencies, such as interdisciplinary teamwork, required in surgery. Furthermore, the use of volunteers in this study may be a source of selection bias toward motivated and technologically savvy learners. Other limitations include the sample cohort with limited surgical experience, instructor experience level, and the remote instruction context that limited in-person expert feedback delivery owing to the COVID-19 pandemic. Whether AI feedback would remain comparable to in-person expert instruction was beyond the scope of this study and is being evaluated by an ongoing trial (ClinicalTrials.gov Identifier: NCT05168150). Future studies should also focus on combining personalized AI feedback with expert instruction to investigate hybrid methods that maximize the educational potential for learners.

## Conclusions

The findings of this randomized clinical trial suggest that performing simulated brain tumor resections was more effective with feedback from an AI tutor compared with learning from remote expert instruction. VOA significantly improved Expertise Scores and OSATS scores in a realistic procedure while fostering an equivalent affective and cognitive learning environment.

## Notes

Supplement 1.
Trial Protocol and Statistical Analysis Plan

Supplement 2. **eFigure 1.** Practice Subpial Tumor Resection Scenario **eFigure 2.** Realistic Subpial Tumor Resection Scenario **eFigure 3.** The Virtual Reality Simulator Platform **eFigure 4.** Learning With the Virtual Operative Assistant (VOA) **eAppendix 1.** Standardized Instructor Training Protocol **eFigure 5.** Remote Expert Instruction **eAppendix 2.** Video Assessment Sheet **eTable.** Intervention Comparison Table **eFigure 6.** Participant’s Progression Through the VOA Training **eFigure 7.** Instructor Group’s OSATS Ratings During Practice

Supplement 3.
Data Sharing Statement

## References

[1] Schlich T. ‘The days of brilliancy are past’: skill, styles and the changing rules of surgical performance, ca. 1820-1920. Med Hist. 2015;59(3):379-403. doi:10.1017/mdh.2015.26 26090735 PMC4597248

[2] Lawrence C. Medical Minds, Surgical Bodies. In: Lawrence C, Shapin S, eds. Science Incarnate: Historical Embodiments of Natural Knowledge. University of Chicago Press; 1998:156-201.

[3] Birkmeyer JD, Finks JF, O’Reilly A, ; Michigan Bariatric Surgery Collaborative. Surgical skill and complication rates after bariatric surgery. N Engl J Med. 2013;369(15):1434-1442. doi:10.1056/NEJMsa1300625 24106936

[4] Stulberg JJ, Huang R, Kreutzer L, . Association between surgeon technical skills and patient outcomes. JAMA Surg. 2020;155(10):960-968. doi:10.1001/jamasurg.2020.3007 32838425 PMC7439214

[5] Rogers MP, DeSantis AJ, Janjua H, Barry TM, Kuo PC. The future surgical training paradigm: virtual reality and machine learning in surgical education. Surgery. 2021;169(5):1250-1252. doi:10.1016/j.surg.2020.09.040 33280858

[6] Davids J, Manivannan S, Darzi A, Giannarou S, Ashrafian H, Marcus HJ. Simulation for skills training in neurosurgery: a systematic review, meta-analysis, and analysis of progressive scholarly acceptance. Neurosurg Rev. 2021;44(4):1853-1867. doi:10.1007/s10143-020-01378-0 32944808 PMC8338820

[7] Reznick R, Harris K, Horsely T, Sheikh Hassani M. Task Force Report on Artificial Intelligence and Emerging Digital Technologies. The Royal College of Physicians and Surgeons of Canada; 2020.

[8] Winkler-Schwartz A, Yilmaz R, Mirchi N, . Machine learning identification of surgical and operative factors associated with surgical expertise in virtual reality simulation. JAMA Netw Open. 2019;2(8):e198363-e198363. doi:10.1001/jamanetworkopen.2019.8363 31373651

[9] Bissonnette V, Mirchi N, Ledwos N, Alsidieri G, Winkler-Schwartz A, Del Maestro RF; Neurosurgical Simulation & Artificial Intelligence Learning Centre. Artificial intelligence distinguishes surgical training levels in a virtual reality spinal task. J Bone Joint Surg Am. 2019;101(23):e127. doi:10.2106/JBJS.18.01197 31800431 PMC7406145

[10] Siyar S, Azarnoush H, Rashidi S, . Machine learning distinguishes neurosurgical skill levels in a virtual reality tumor resection task. Med Biol Eng Comput. 2020;58(6):1357-1367. doi:10.1007/s11517-020-02155-3 32279203

[11] Munro C, Burke J, Allum W, Mortensen N. COVID-19 leaves surgical training in crisis. BMJ. 2021;372(n659):n659. doi:10.1136/bmj.n659 33712499

[12] Tomlinson SB, Hendricks BK, Cohen-Gadol AA. Editorial. Innovations in neurosurgical education during the COVID-19 pandemic: is it time to reexamine our neurosurgical training models? J Neurosurg. 2020;133(1):1-2. doi:10.3171/2020.4.JNS201012 32302991 PMC7164321

[13] Ma W, Adesope OO, Nesbit JC, Liu Q. Intelligent tutoring systems and learning outcomes: a meta-analysis. J Educ Psychol. 2014;106(4):901-918. doi:10.1037/a0037123

[14] Mirchi N, Bissonnette V, Yilmaz R, Ledwos N, Winkler-Schwartz A, Del Maestro RF. The virtual operative assistant: an explainable artificial intelligence tool for simulation-based training in surgery and medicine. PLoS One. 2020;15(2):e0229596. doi:10.1371/journal.pone.0229596 32106247 PMC7046231

[15] Harris KA, Nousiainen MT, Reznick R. Competency-based resident education—the Canadian perspective. Surgery. 2020;167(4):681-684. doi:10.1016/j.surg.2019.06.033 31431292

[16] van Merriënboer JJG, Kester L. The Four-Component Instructional Design Model: Multimedia Principles in Environments for Complex Learning. In: Mayer RE, ed. The Cambridge Handbook of Multimedia Learning. 2nd ed. Cambridge University Press; 2014:104-148. doi:10.1017/CBO9781139547369.007

[17] Chao TN, Frost AS, Brody RM, . Creation of an interactive virtual surgical rotation for undergraduate medical education during the COVID-19 pandemic. J Surg Educ. 2021;78(1):346-350. doi:10.1016/j.jsurg.2020.06.039 32654999 PMC7328635

[18] Rojas-Muñoz E, Cabrera ME, Lin C, . The System for Telementoring with Augmented Reality (STAR): a head-mounted display to improve surgical coaching and confidence in remote areas. Surgery. 2020;167(4):724-731. doi:10.1016/j.surg.2019.11.008 31916990

[19] Butt KA, Augestad KM. Educational value of surgical telementoring. J Surg Oncol. 2021;124(2):231-240. doi:10.1002/jso.26524 34245572 PMC8361692

[20] Martin JA, Regehr G, Reznick R, . Objective structured assessment of technical skill (OSATS) for surgical residents. Br J Surg. 1997;84(2):273-278. 9052454 10.1046/j.1365-2168.1997.02502.x

[21] Eppich W, Cheng A. Promoting Excellence and Reflective Learning in Simulation (PEARLS): development and rationale for a blended approach to health care simulation debriefing. Simul Healthc. 2015;10(2):106-115. doi:10.1097/SIH.0000000000000072 25710312

[22] Liu X, Cruz Rivera S, Moher D, Calvert MJ, Denniston AK; SPIRIT-AI and CONSORT-AI Working Group. Reporting guidelines for clinical trial reports for interventions involving artificial intelligence: the CONSORT-AI extension. Lancet Digit Health. 2020;2(10):e537-e548. doi:10.1016/S2589-7500(20)30218-1 33328048 PMC8183333

[23] Winkler-Schwartz A, Bissonnette V, Mirchi N, . Artificial intelligence in medical education: best practices using machine learning to assess surgical expertise in virtual reality simulation. J Surg Educ. 2019;76(6):1681-1690. doi:10.1016/j.jsurg.2019.05.015 31202633

[24] Random.org. Accessed January 24, 2022. https://www.random.org/

[25] Duffy MC, Lajoie SP, Pekrun R, Lachapelle K. Emotions in medical education: Examining the validity of the Medical Emotion Scale (MES) across authentic medical learning environments. Learn Instr. 2020;70:101150. doi:10.1016/j.learninstruc.2018.07.001

[26] Lynch J, Aughwane P, Hammond TM. Video games and surgical ability: a literature review. J Surg Educ. 2010;67(3):184-189. doi:10.1016/j.jsurg.2010.02.010 20630431

[27] Rui M, Lee JE, Vauthey J-N, Conrad C. Enhancing surgical performance by adopting expert musicians’ practice and performance strategies. Surgery. 2018;163(4):894-900. doi:10.1016/j.surg.2017.09.011 29336812

[28] Macnamara BN, Moreau D, Hambrick DZ. The relationship between deliberate practice and performance in sports: a meta-analysis. Perspect Psychol Sci. 2016;11(3):333-350. doi:10.1177/1745691616635591 27217246

[29] Bugdadi A, Sawaya R, Bajunaid K, . Is virtual reality surgical performance influenced by force feedback device utilized? J Surg Educ. 2019;76(1):262-273. doi:10.1016/j.jsurg.2018.06.012 30072262

[30] Sabbagh AJ, Bajunaid KM, Alarifi N, . Roadmap for developing complex virtual reality simulation scenarios: subpial neurosurgical tumor resection model. World Neurosurg. 2020;139:e220-e229. doi:10.1016/j.wneu.2020.03.187 32289510

[31] Leppink J, Paas F, Van der Vleuten CPM, Van Gog T, Van Merriënboer JJG. Development of an instrument for measuring different types of cognitive load. Behav Res Methods. 2013;45(4):1058-1072. doi:10.3758/s13428-013-0334-1 23572251

[32] Delorme S, Laroche D, DiRaddo R, Del Maestro RF. NeuroTouch: a physics-based virtual simulator for cranial microneurosurgery training. Neurosurgery. 2012;71(1)(Suppl Operative):32-42. doi:10.1227/NEU.0b013e318249c744 22233921

[33] Gélinas-Phaneuf N, Choudhury N, Al-Habib AR, . Assessing performance in brain tumor resection using a novel virtual reality simulator. Int J Comput Assist Radiol Surg. 2014;9(1):1-9. doi:10.1007/s11548-013-0905-8 23784222

[34] Yilmaz R, Winkler-Schwartz A, Mirchi N, Reich A, Del Maestro R. O51: artificial intelligence utilizing recurrent neural networks to continuously monitor composites of surgical expertise. Br J Surg. 2021;108(suppl 1):znab117. doi:10.1093/bjs/znab117.051

[35] Hebb AO, Yang T, Silbergeld DL. The sub-pial resection technique for intrinsic tumor surgery. Surg Neurol Int. 2011;2:180. doi:10.4103/2152-7806.90714 22368786 PMC3267372

[36] Zimmerman BJ. Investigating Self-Regulation and Motivation: Historical Background, Methodological Developments, and Future Prospects. Am Educ Res J. 2008;45(1):166-183. doi:10.3102/0002831207312909

[37] McGaghie WC. Mastery learning: it is time for medical education to join the 21st century. Acad Med. 2015;90(11):1438-1441. doi:10.1097/ACM.0000000000000911 26375269

[38] Ericsson KA, Hoffman RR, Kozbelt A, Williams AM, eds. The Cambridge Handbook of Expertise and Expert Performance. Cambridge University Press; 2018. doi:10.1017/9781316480748

[39] Winkler-Schwartz A, Marwa I, Bajunaid K, . A comparison of visual rating scales and simulated virtual reality metrics in neurosurgical training: a generalizability theory study. World Neurosurg. 2019;127:e230-e235. doi:10.1016/j.wneu.2019.03.059 30880209

[40] Kolb DA. Experiential Learning: Experience as the Source of Learning and Development. FT Press; 2014.

[41] Fecso AB, Szasz P, Kerezov G, Grantcharov TP. The effect of technical performance on patient outcomes in surgery: a systematic review. Ann Surg. 2017;265(3):492-501. doi:10.1097/SLA.0000000000001959 27537534

[42] Dean WH, Gichuhi S, Buchan JC, . Intense simulation-based surgical education for manual small-incision cataract surgery: the Ophthalmic Learning and Improvement Initiative in Cataract Surgery Randomized Clinical Trial in Kenya, Tanzania, Uganda, and Zimbabwe. JAMA Ophthalmol. 2021;139(1):9-15. doi:10.1001/jamaophthalmol.2020.4718 33151321 PMC7645744

[43] Meling TR, Meling TR. The impact of surgical simulation on patient outcomes: a systematic review and meta-analysis. Neurosurg Rev. 2021;44(2):843-854. doi:10.1007/s10143-020-01314-2 32399730 PMC8035110

[44] Lohre R, Bois AJ, Athwal GS, Goel DP; Canadian Shoulder and Elbow Society (CSES). Improved complex skill acquisition by immersive virtual reality training: a randomized controlled trial. J Bone Joint Surg Am. 2020;102(6):e26. doi:10.2106/JBJS.19.00982 31972694

[45] Issenberg SB, McGaghie WC, Petrusa ER, Lee Gordon D, Scalese RJ. Features and uses of high-fidelity medical simulations that lead to effective learning: a BEME systematic review. Med Teach. 2005;27(1):10-28. doi:10.1080/01421590500046924 16147767

[46] Kaufman DM, Mann KV. Teaching and Learning in Medical Education: How Theory can Inform Practice. Postgrad Med J. 2001;77:551. doi:10.1136/pmj.77.910.551c

[47] Bouchet F, Harley JM, Azevedo R. Evaluating adaptive pedagogical agents’ prompting strategies effect on students’ emotions. Paper presented at: 14th International Conference on Intelligent Tutoring Systems; June 11, 2018; Montreal, Canada.

[48] Harley JM, Bouchet F, Azevedo R. Aligning and comparing data on emotions experienced during learning with MetaTutor. In: Lane HC, Yacef K, Mostow J, Pavlik P, eds. Artificial Intelligence in Education. AIED 2013. Lecture Notes in Computer Science. Springer; 2013. doi:10.1007/978-3-642-39112-5_7

[49] Schaffir J, Strafford K, Worly B, Traugott A. Challenges to medical education on surgical services during the COVID-19 pandemic. Med Sci Educ. 2020;30(4):1-5. doi:10.1007/s40670-020-01072-2 32904384 PMC7455508

[50] Mirchi N, Ledwos N, Del Maestro RF. Intelligent tutoring systems: re-envisioning surgical education in response to COVID-19. Can J Neurol Sci. 2021;48(2):198-200, doi:10.1017/cjn.2020.202 32907644 PMC7642506

[51] Anderson LW. Curricular alignment: a re-examination. Theory Pract. 2002;41(4):255-260. doi:10.1207/s15430421tip4104_9

[52] Biggs J. Enhancing teaching through constructive alignment. Higher Ed. 1996;32(3):347-364. doi:10.1007/BF00138871

[53] Eppich WJ, Hunt EA, Duval-Arnould JM, Siddall VJ, Cheng A. Structuring feedback and debriefing to achieve mastery learning goals. Acad Med. 2015;90(11):1501-1508. doi:10.1097/ACM.0000000000000934 26375272

[54] Janzen KJ, Jeske S, MacLean H, . Handling strong emotions before, during, and after simulated clinical experiences. Clin Simul Nurs. 2016;12(2):37-43. doi:10.1016/j.ecns.2015.12.004

[55] Bilgic E, Turkdogan S, Watanabe Y, . Effectiveness of telementoring in surgery compared with on-site mentoring: a systematic review. Surg Innov. 2017;24(4):379-385. doi:10.1177/1553350617708725 28494684

[56] Erridge S, Yeung DKT, Patel HRH, Purkayastha S. Telementoring of surgeons: a systematic review. Surg Innov. 2019;26(1):95-111. doi:10.1177/1553350618813250 30465477

[57] Levin M, McKechnie T, Kruse CC, Aldrich K, Grantcharov TP, Langerman A. Surgical data recording in the operating room: a systematic review of modalities and metrics. Br J Surg. 2021;108(6):613-621. doi:10.1093/bjs/znab016 34157080

