# Supplementary material for: Effect of Artificial Intelligence Tutoring vs Expert Instruction on Learning Simulated Surgical Skills Among Medical Students: A Randomized Clinical Trial
Source: JAMA Netw Open. 2022 Feb 22;5(2):e2149008. doi: 10.1001/jamanetworkopen.2021.49008 (PMC8864513; doi:10.1001/jamanetworkopen.2021.49008)
Supplement: Supplement 1. — Trial Protocol and Statistical Analysis Plan [file jamanetwopen-e2149008-s001.pdf]

Study Protocol and Statistical Analysis Plan

Effectiveness of an Artificial Intelligent Tutoring System in Simulation Training

ClinicalTrials.gov Identifier: NCT04700384

Recruitment Status: Completed
First Posted: January 7, 2021
Last Update Posted: May 24, 2021

Sponsor: McGill University
Information Provided by (Responsible Party):
Rolando F. Del Maestro, McGill University

Study Description

Brief Summary:

Background:
Although surgical experience and technical skill are associated with better patient outcomes [1-3], quantitating surgical ability in the operating room is challenging. In surgical education, large datasets generated by high-fidelity virtual reality simulators can be employed by machine learning algorithms to objectively measure trainee performance and competence on expert benchmarks[4]. This allows repetitive practice of surgical skills in safe and risk-free environments with immediate feedback.

Our group developed and has a patent pending for an intelligent tutoring system called the Virtual Operative Assistant (VOA) [5,6]. Utilizing a support vector machine algorithm, the VOA

assesses data derived from the NeuroVR (CAE Healthcare) simulator platform and provides individualized audiovisual feedback to improve learner performance during simulated brain tumor resections. The effectiveness of intelligent tutoring systems such as the VOA to the human surgical apprenticeship pedagogy remains to be elucidated.

The aim of this study is to compare the effectiveness and educational impact of personalized VOA feedback to expert instruction on medical student's technical skills learning of a virtual reality tumor resection procedure.

Specific Aims: 1) To assess if medical students receiving personalized VOA feedback statistically improve their surgical performance when compared to those having (a) no expert instructor feedback or (b) expert instructor-mediated feedback. 2) To outline if different emotions are elicited by the VOA intelligent tutoring system in medical students while performing this achievement task as compared to human instruction.

Design: A three-arm partially blinded randomized controlled trial of VOA training versus remote-based expert instruction versus control.

Setting: Neurosurgical Simulation and Artificial Intelligence Learning Centre, Montreal Neurological Institute.

Participants: Eligible Medicine-Preparatory (Med-P), first- and second-year medical students from across the province of Quebec.

Task: Complete removal of a simulated tumour – distinguishable by colour and haptic properties – with minimal bleeding and damage to surrounding healthy brain using two surgical instruments (Cavitron Ultrasonic Aspirator and Bipolar pincers) of the NeuroVR (CAE Healthcare) surgical simulator.

Intervention: A single 75-minute training session, including six virtual subpial tumour resection attempts (five simple practice scenarios and one complex realistic scenario) with assessment and feedback from either:

1) the VOA intelligent tutoring system (Group 2) or

2) a remote-based expert instructor (Group 3)

Both compared to:

3) control group (Group 1) that receives no assessment or performance feedback.

Main Outcomes and Measures:

Primary outcome is surgical performance improvement and learning. Performance improvement is measured as the difference between participant's attempts during the five practice attempts along four performance metric categories (Safety, Efficiency, Quality and Bimanual Cognitive) recorded by the simulator, assessed, classified and presented by previously established machine learning algorithms [4]. Learning is measured by the participant's composite performance score (obtained from blinded instructor assessment of videos and the simulator's assessment, both weighting 50%) on their performance of the more complex and realistic tumour resection scenario.

Secondary outcomes include the strength of emotions and level of cognitive load experienced by participants in each intervention arm, assessed through three questionnaires sets before, during and after the training intervention.

To our knowledge this will be the first study to compare the effectiveness of an AI-powered intelligent tutoring system to expert instruction in the context of medical and surgical virtual reality simulation and assess the emotional response to such instruction. This study aims to begin to identify successful approaches to use this innovative technology in the medical educational curriculum and improve patient outcomes by augmenting safety, efficiency and competency of surgeons and other healthcare providers.

Condition or disease	Intervention/treatment	Phase
Surgical Education	Behavioral: Virtual Operative Assistant Training<br><br>Behavioral: Remote-Based Expert Instructor Training	Not Applicable

Detailed Description:

Background

The advent of the ongoing COVID-19 pandemic has impacted medical education by limiting students' access to hands-on surgical and clinical training. With uncertainty over how clerkship rotations are going to look, research on novel educational tools is required to offer solutions that prevent a gap in the learner's technical competence. Technological advancements in the fields of virtual reality (VR) and artificial intelligence (AI), combined with a shift toward competency-based medical education have resulted in the development and validation of surgical simulators for residency training [4]. VR simulators can supplement the traditional apprenticeship-based model of training for surgeons, and other healthcare providers that depend on technical expertise, by providing learners with unlimited opportunities for repetitive practice in a risk-free environment. This would allow students to practice surgical and other technical skills without the limitations imposed by the need for supervision, case availability or learning environments. Simulators record an enormous amount of data during VR task performance. These datasets provide novel insights into surgical expertise, offer real-time procedural guidance, give automated feedback, and inform educators in developing objective assessment measures [4-6].

Rationale

A series of AI algorithms that accurately assess bimanual psychomotor surgical performance in a virtual subpial tumor resection task have been assessed and validated [5] and a patent for these systems, called the Virtual Operative Assistant (VOA), has recently been published (PCT/CA2020/050353, Title: A Framework For Transparent Artificial Intelligence In Simulation: The Virtual Operative Assistant, (WO 2020/186348) published in September 24, 2020) [6]. The

Virtual Operative Assistant involves software that uses machine learning algorithms and artificial neural networks to provide learners with personalized formative feedback based on their performance during a simulated neurosurgical procedure [5]. This AI-powered tutoring system analyses large multimodal data regarding an individual's bimanual psychomotor skills, compares it with data from an expert's performance in that task and suggests areas for improvement. However, the effectiveness of the VOA in improving surgical learning and performance compared to the traditional instructor-based method is not known.

Research Objectives

To compare the effectiveness of personalized VOA feedback to expert instruction on medical student's learning outcomes during simulated VR tumor resection procedures and examine which educational intervention results in: 1) better surgical performance and learning outcome (primary outcome) and 2) lower cognitive load and the activation of positively valence emotions that can support learning (secondary outcome).

Hypothesis

VOA feedback will 1) significantly improve medical student's performance compared to no feedback, 2) be non-inferior to expert-mediated feedback and 3) result in lower negative valence emotions.

Study Design

Study Type:	Interventional
Enrollment:	72 participants
Allocation:	Parallel Assignment<br>Randomized, partially blinded
Masking:	Double (Participant, Outcomes Assessor)<br><br>Participants do not know the performance metrics used in their final evaluation, only that they will be learning and practicing technical skills used in neurosurgery for subpial tumor resection procedures.

	All participants are told that their on-screen performance is being observed by an expert in a different room.<br><br>Instructors assessing the performance videos for the realistic scenario are blinded to the participant's group assignment.
Primary Purpose:	Health Services Research
Official Title:	Comparing the Virtual Operative Assistant to Instructor-based Instruction in Surgical Education: A Randomized Controlled Trial
Actual Study Start Date:	January 15, 2021
Actual Primary Completion Date:	May 15, 2021
Actual Study Completion Date:	May 15, 2021

Arms and Interventions

Arm	Intervention/treatment
Control Group – Baseline Training<br><br>23 Participants allocated. Individuals receive introductory information on using the simulator and the scenario. They perform 5 simple subpial tumour resections for practice and have 5 minutes per trial. After each attempt, the student takes a 5-minute break with no assessment or feedback on their performance. On their 6th attempt they have 13 minutes to perform a different realistic scenario.	No assessment or feedback.<br><br>The average performance improvement by this group will determine the baseline for learning possible with only using the simulator.
Experimental Group – VOA Training<br><br>23 participants allocated. Individuals receive the same information, have the same amount of time and perform the same scenarios as the control group. In the 5-	VOA Assessment and Feedback.<br><br>Students receive a percentage score of their performance based on their level of expertise in four performance metrics determined by the system's support vector machine. If the

minutes between attempts, participant receive the VOA's assessment of their performance and audiovisual feedback.	performance is outside the expert reference benchmark in a given metric, participants observe a feedback video which demonstrates an expert performance and provides constructive directional feedback to excel.
Experimental Group – remote-based expert Instructor Training<br><br>23 participants allocated. Individuals receive the same information, have the same amount of time and perform the same scenarios as the control group. Meanwhile, a trained instructor observes the participant's on-screen performance, that is live-streamed, remotely. Instructors are senior neurosurgery residents with extensive experience in performing and assessing this scenario. During the 5-minute feedback session, they chat with the student, discussing the performance and help in setting goals for the next trial.<br><br>NOTE: To control for Hawthorne (observer) effect, all participants are told that their performance is being streamed to an instructor.	Remote-based expert instructor assessment and feedback.<br><br>As the participant performs the simulation, instructors grade the on-screen performance using a previously established an Objective Structured Assessment of Technical Skills (OSATS) Visual Rating Scale in six components (e.g., Respect for Tissue, Flow, and Efficiency of Movement) [7] on a 7-point Likert scale. During the feedback session, instructors select feedback statements from a standardized list, discuss the participant's areas of improvement and help them set specific goals for the next attempt.

Outcome Measures

Primary Outcome Measure:

1. Change in procedural performance

Performance in each practice attempt is measured utilizing raw data from the simulator that is used for assessment by previously established AI algorithms on validated metrics (ICEMS Expertise Score).

2. Change in learning

Performance on the complex realistic scenario is evaluated by expert instructors using the OSATS Visual Rating Scale and the AI assessment algorithms (ICEMS Expertise Score).

Secondary Outcome Measure:

1. Difference in the strength of emotions elicited

Measured using Duffy's Medical Emotions Scale (MES) [8], before, during and after the intervention.

2. Difference in cognitive load

Measured using Leppink's Cognitive Load Index (CLI) [9] after the intervention.

Eligibility Criteria

Ages Eligible for Study:	18 Years and older
Sexes Eligible for Study:	All
Gender Based:	No
Accepts Healthy Volunteers:	Yes

Criteria

Inclusion Criteria:

- First- and second-year medical students from any Canadian institution who do not meet the exclusion criteria.

Exclusion Criteria:

- Participation in our group's previous trials involving the NeuroVR (CAE Healthcare) simulator.
- Prior surgical clerkship experience.

Contacts and Locations

Locations

Canada, Quebec

Neurosurgical Simulation and Artificial Intelligence Learning Centre,
Montreal Neurological Institute,
McGill University, Montreal, H3A 2B4
Quebec, Canada

Investigators

Principal Investigator:	Rolando F. Del Maestro, MD PhD	McGill University

More Information

Responsible Party:	Rolando F. Del Maestro, William Feindel Professor Emeritus in Neuro-Oncology, McGill University
ClinicalTrials.gov Identifier:	NCT04700384
Human Subjects Ethics Review Board Status:	Approved
Ethics Review Board Project Number:	2010-270, NEU-09-042
Studies a U.S. FDA-regulated Drug Product:	No
Studies a U.S. FDA-regulated Device Product:	No

Statistical Analysis Plan

To achieve a statistical power of 0.80, considering a potential effect size of 35% and a significance level of 0.05, this study requires a minimum of 23 participants in each group. All collected participant data will be anonymized and kept in a locked cabinet. Participant characteristics are described as count with percentages, mean (SD) or median (IQR), as appropriate. Validated artificial intelligence algorithms will analyze raw performance data and evaluate the participant's performance utilizing previous established competence benchmarks for practice and realistic tumor resection scenarios.

Performance videos will be scored by blinded experts using previously published visual rating scales. Continuous data will be checked for outliers and tests of normality, sphericity, and homogeneity of variance will be conducted to check the assumptions of ANOVA. Emotions before, during, and after, and procedural performance in the 5 practice resections will be examined by a two-way mixed ANOVA using time as the within-subjects variable and group

allocation as the between-subjects variable. Baseline performance (i.e., performance in the first practice subpial resection) will be treated as a covariate in the mixed model for procedural performance analysis. Post-intervention responses to the CLI will be summarized for each group and evaluated using one-way ANOVA. Before recruitment, inter-rater reliability will be evaluated using intraclass correlation coefficient and OSATS scale consistency will be examined using Cronbach's alpha from data gathered from instructor training.

All statistical tests will be conducted in SPSS version 27 (IBM Corporation, 2020 release, Armonk, New York, United States). Expertise Score predictions were conducted in MATLAB R2020a release (MathWorks Inc., Natick, Massachusetts, United States).

References:

1. Birkmeyer JD, Finks JF, O'Reilly A, et al. Surgical Skill and Complication Rates after Bariatric Surgery. *New England Journal of Medicine.* 2013;369(15):1434-1442.
2. Birkmeyer JD, Stukel TA, Siewers AE, Goodney PP, Wennberg DE, Lucas FL. Surgeon Volume and Operative Mortality in the United States. *New England Journal of Medicine.* 2003;349(22):2117-2127.
3. Stulberg JJ, Huang R, Kreutzer L, et al. Association Between Surgeon Technical Skills and Patient Outcomes. *JAMA Surgery.* 2020;155(10):960-968.
4. Winkler-Schwartz A, Yilmaz R, Mirchi N, et al. Machine Learning Identification of Surgical and Operative Factors Associated With Surgical Expertise in Virtual Reality Simulation. *JAMA Network Open.* 2019;2(8):e198363-e198363.
5. Mirchi N, Bissonnette V, Yilmaz R, Ledwos N, Winkler-Schwartz A, Del Maestro RF. The Virtual Operative Assistant: An explainable artificial intelligence tool for simulation-based training in surgery and medicine. *PLOS ONE.* 2020;15(2):e0229596.
6. Mirchi N, Yilmaz R, Winkler-Schwartz A, Bissonnette V, Ledwos N, Del Maestro R, Inventors. A FRAMEWORK FOR TRANSPARENT ARTIFICIAL INTELLIGENCE IN SIMULATION: THE VIRTUAL OPERATIVE ASSISTANT. 24 September 2020, 2020.
7. Winkler-Schwartz A, Marwa I, Bajunaid K, et al. A Comparison of Visual Rating Scales and Simulated Virtual Reality Metrics in Neurosurgical Training: A Generalizability Theory Study. *World Neurosurgery.* 2019;127:e230-e235.
8. Duffy MC, Lajoie SP, Pekrun R, Lachapelle K. Emotions in medical education: Examining the validity of the Medical Emotion Scale (MES) across authentic medical learning environments. *Learning and Instruction.* 2020;70:101150.
9. Leppink J, Paas F, Van der Vleuten CPM, Van Gog T, Van Merriënboer JJG. Development of an instrument for measuring different types of cognitive load. *Behavior Research Methods.* 2013;45(4):1058-1072.
