# Supplementary material for: Effect of Artificial Intelligence Tutoring vs Expert Instruction on Learning Simulated Surgical Skills Among Medical Students: A Randomized Clinical Trial
Source: JAMA Netw Open. 2022 Feb 22;5(2):e2149008. doi: 10.1001/jamanetworkopen.2021.49008 (PMC8864513; doi:10.1001/jamanetworkopen.2021.49008)
Supplement: Supplement 2. — eFigure 1. Practice Subpial Tumor Resection Scenario eFigure 2. Realistic Subpial Tumor Resection Scenario eFigure 3. The Virtual Reality Simulator Platform eFigure 4. Learning With the Virtual Operative Assistant (VOA) eAppendix 1. Standardized Instructor Training Protocol eFigure 5. Remote Expert Instruction eAppendix 2. Video Assessment Sheet eTable. Intervention Comparison Table eFigure 6. Participant’s Progression Through the VOA Training eFigure 7. Instructor Group’s OSATS Ratings During Practice [file jamanetwopen-e2149008-s002.pdf]

Supplemental Online Content

Fazlollahi AM, Bakhaidar M, Alsayegh A, et al. Effect of artificial intelligence tutoring vs expert instruction on learning simulated surgical skills among medical students: a randomized clinical trial. *JAMA Netw Open*. 2022;5(2):e2149008. doi:10.1001/jamanetworkopen.2021.49008

eFigure 1. Practice Subpial Tumor Resection Scenario
eFigure 2. Realistic Subpial Tumor Resection Scenario
eFigure 3. The Virtual Reality Simulator Platform
eFigure 4. Learning With the Virtual Operative Assistant (VOA)
eAppendix 1. Standardized Instructor Training Protocol
eFigure 5. Remote Expert Instruction
eAppendix 2. Video Assessment Sheet
eTable. Intervention Comparison Table
eFigure 6. Participant's Progression Through the VOA Training
eFigure 7. Instructor Group's OSATS Ratings During Practice

This supplemental material has been provided by the authors to give readers additional information about their work.

© 2022 Fazlollahi AM et al. *JAMA Network Open.*

eFigure 1. Practice Subpial Tumor Resection Scenario

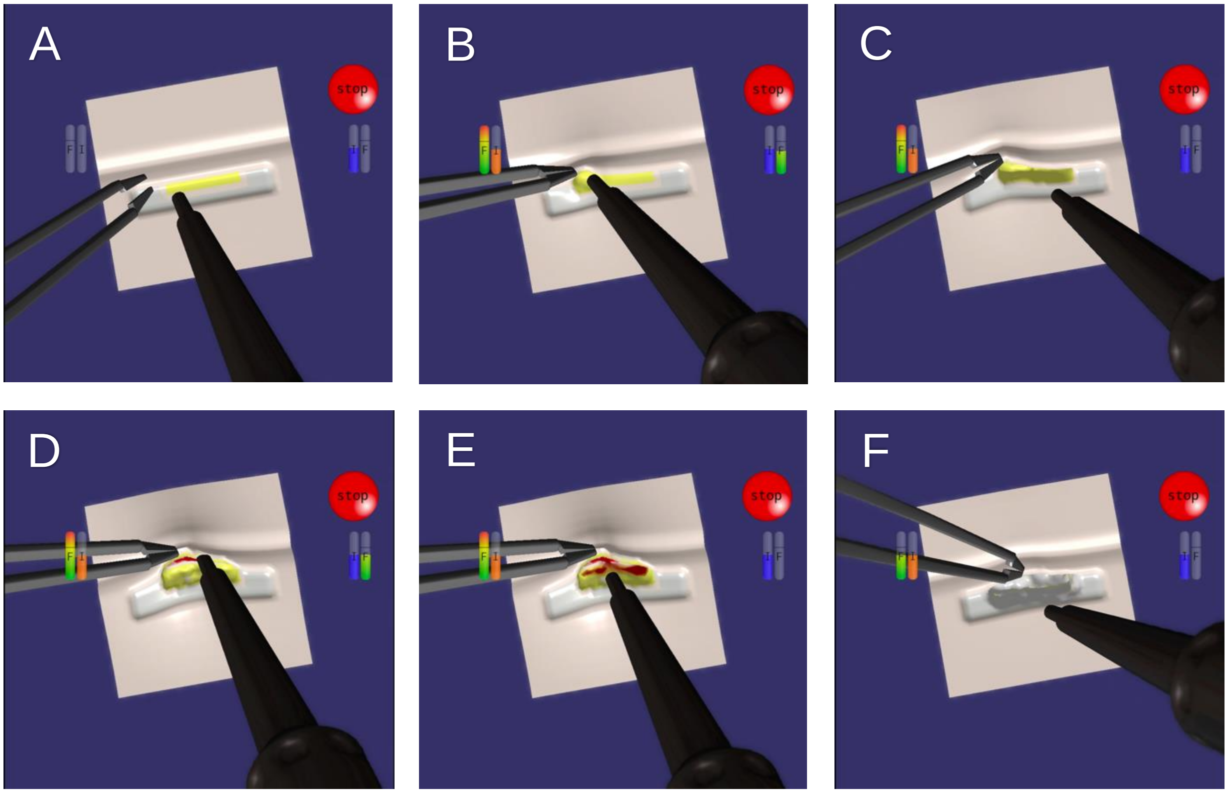

(A) Practice subpial scenario at the start of the simulation, yellow tissue represents the tumor, instrument on the left is the bipolar and the instrument on the right is the aspirator. **(B)** Participant using the bipolar to lift the pia and aspirator utilized to resect the tumor lying beneath the pia. **(C)** Appearance following resection of superficial tumor. Yellow tissue remaining depicts the deeper tumor areas. **(D)** Participant exposing the simulated deep cerebral vessel (red). **(E)** Instrument injury to the blood vessel resulting in bleeding. **(F)** Complete resection of the tumor.

© 2022 Fazlollahi AM et al. *JAMA Network Open.*

eFigure 2. Realistic Subpial Tumor Resection Scenario

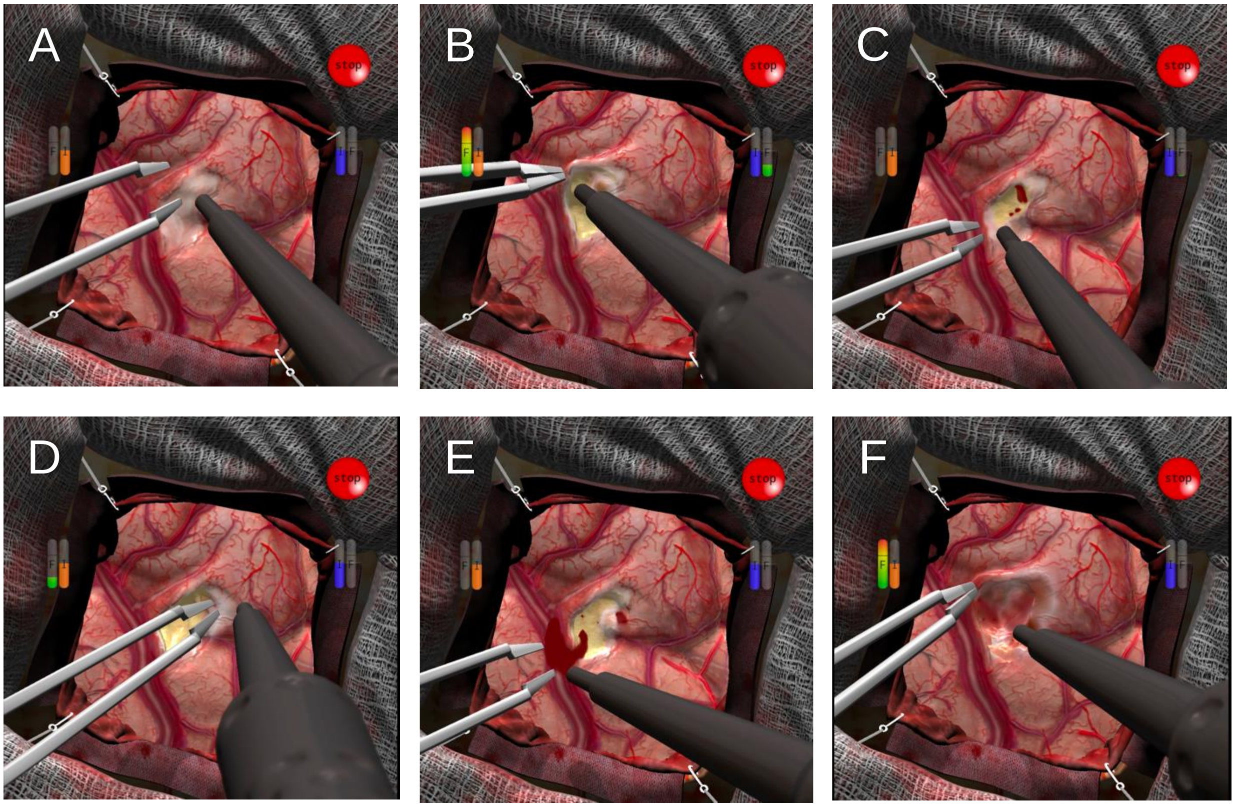

(A) Realistic subpial scenario at the start of the simulation, off-white tissue represents the tumor. **(B)** Participant using the simulated bipolar to lift pia and begins resecting tumor with the simulated aspirator. **(C)** Participant while using the aspirator causes minor bleeding from the tumor. **(D)** Participant cauterizing bleeding points. **(E)** Injury to the superficial cerebral vein followed by significant bleeding. **(F)** Completed Tumor resection.

© 2022 Fazlollahi AM et al. *JAMA Network Open.*

eFigure 3. The Virtual Reality Simulator Platform

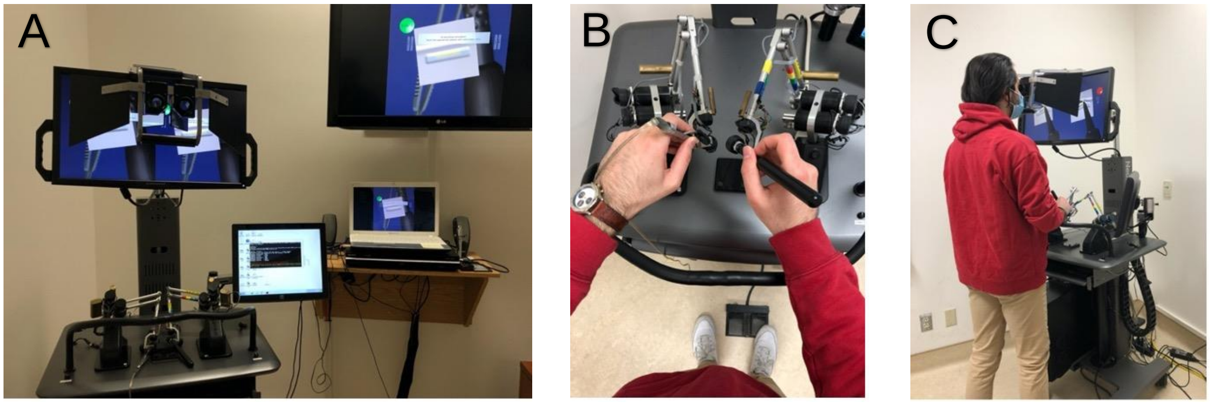

(A) The NeuroVR simulator with the practice subpial scenario on the screen. **(B)** Participant using the handles for subpial tumor resection (bipolar instrument with the left hand and the aspirator with the right hand) foot pedals (at the bottom of the image) control the activation of the corresponding instruments. **(C)** Participant viewing the screen through the stereoscope and performing the practice subpial scenario.

© 2022 Fazlollahi AM et al. *JAMA Network Open.*

eFigure 4. Learning With the Virtual Operative Assistant (VOA)

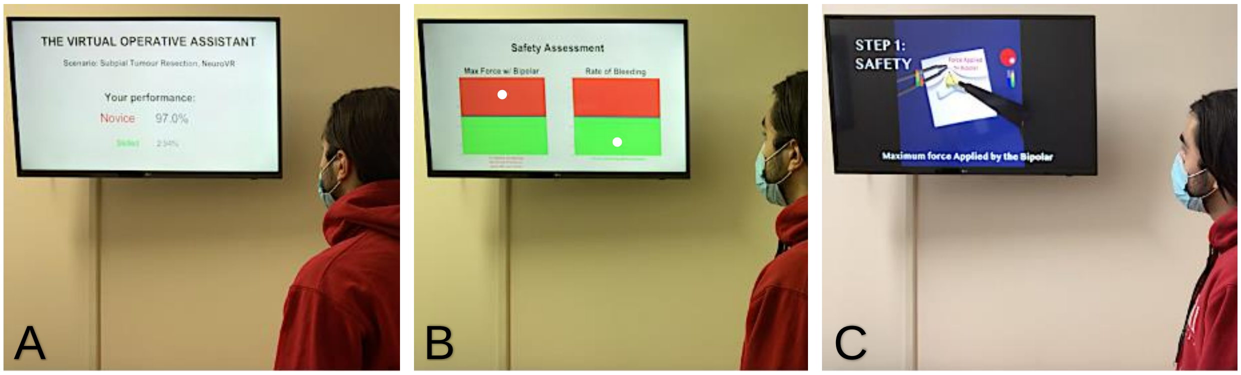

(A) Participant viewing the VOA's performance prediction of their practice subpial resection. **(B)** Participant viewing a breakdown of their performance assessment on two safety metrics. A score in the red box (depicted by the white dot) suggests falling outside the competence benchmark for that metric. **(C)** VOA plays the appropriate feedback video for the metric that needs improvement.

© 2022 Fazlollahi AM et al. *JAMA Network Open.*

eAppendix 1. Standardized Instructor Training Protocol

Objective:
To adapt the traditional apprenticeship learning model to a remote context, we need to ensure that the study instructors are trained to:

1. Perform the simulated practice and realistic subpial resections expertly
2. Rate students' performance from screen-recorded videos with consistency and reliability
3. Deliver constructive feedback in scripted debriefing sessions.

Methods:
Eight 90-minute learning sessions in a two-week workshop, provided two senior neurosurgery resident instructors (A.A., M.B., Post Graduate Year 5) with standardized training to become proficient at leading virtual pedagogical sessions remotely for medical student participants of this study.

Two sessions involved performing the simulated resections under the supervision of a senior consultant, who demonstrated the technical competencies required, explained OSATS's qualitative assessment criteria, and described how to lead an effective debriefing based on the PEARLS model.

In the following sessions, instructors trained independently through deliberate practice guided by self-regulated learning where they graded their own screen-recorded performance using the Assessment Sheet (eMethod 2).

Since traditional apprenticeship experts in the operating room have no access to performance metrics and assessment depends only on visual rating, instructors were blinded and unaware of the AI assessment metrics to best replicate the current intraoperative instruction and reduce potential bias in their assessment and instruction in the study.

At the end of the training, instructors were evaluated by the senior consultant based on their ability to achieve technical competence in both simulation resections and lead scripted debriefing sessions remotely. Scale consistency and inter-rater reliability was determined from instructor ratings of 20 randomly selected videos of medical students' performance of both realistic and practice subpial simulations.

Theories used in training:
This training utilized two key educational theories: deliberate practice and self-regulated learning (SRL). Both deliberate practice and SRL accelerated learning by leveraging effective learning strategies, such as drawing upon reflective observation through self-assessment and using forethought to set specific performance goals.

eFigure 5. Remote Expert Instruction

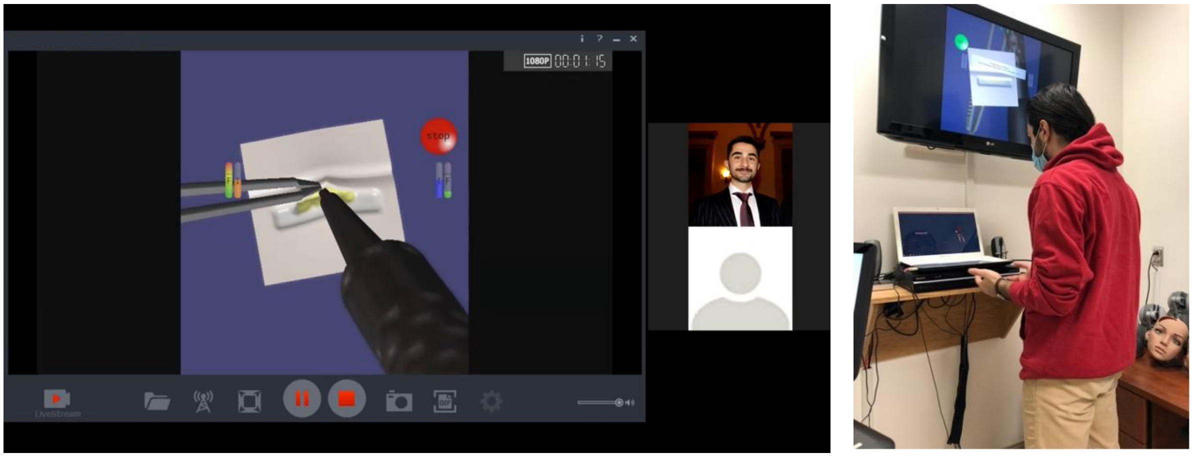

Left. Livestream on-screen performance of a participant's practice resection shared virtually with an instructor located remotely. **Right.** Participant debriefing and receiving feedback from the remote instructor after the simulation resection through a videotelephony software.

© 2022 Fazlollahi AM et al. *JAMA Network Open.*

eAppendix 2. Video Assessment Sheet

Initials (rater): Date:
Subpial Scenario: Practice / Realistic

<u>Video Number:</u>

What did the participant do well?	Identify up to three areas of improvement for this participant:	List two instructions/feedback you would give to this participant:
	1.<br><br>2.<br><br>3.	1.<br><br>2.

OSATS Visual Rating – 7-point Likert Scale

Instrument Handling: how would you rate this participant's ability to handle instruments appropriately and make fluid movements?

Novice 1 2 3 4 5 6 7 Expert

Respect for Tissue: what is the level of care this participant shows for the tissue and the surrounding brain?

Novice 1 2 3 4 5 6 7 Expert

Hemostasis: How would you rate this participant's ability to control bleeding? If no bleeding occurred write N/A.

Novice 1 2 3 4 5 6 7 Expert

Economy of Movement: How would you rate this participant's efficiency of movement?

Novice 1 2 3 4 5 6 7 Expert

Flow: How would you rate this participant's flow of movement in the operation?

Novice 1 2 3 4 5 6 7 Expert

Overall: How would you rate this participant's overall performance in removing a considerable amount of the tumor competently?

Novice 1 2 3 4 5 6 7 Expert

© 2022 Fazlollahi AM et al. *JAMA Network Open.*

eTable. Intervention Comparison Table

	Virtual Operative Assistant	Remote Expert Instruction
**Task Goal**	Complete resection of the tumor with minimal bleeding and damage to healthy tissues.	
**Learning Objectives (Competency Criteria)**	**Safety:**<br>1. Maximum force applied with the bipolar forceps<br>2. Mean rate of bleeding<br>**Movement:**<br>3. Mean instrument tip separation<br>4. Mean Acceleration of the bipolar forceps	**Safety:**<br>1. Respect for Tissue<br>2. Hemostasis<br>**Movement:**<br>3. Instrument Handling<br>4. Economy of Movement<br>5. Flow<br>**Overall Quality:**<br>6. Overall
**Performance Assessment Tool**	Criteria-based assessment using a machine learning classifier algorithm. Four AI-selected metrics used by a support vector machine for performance classification and quantitative benchmark evaluation.	Criteria-based assessment using the Video Assessment Sheet (eMethod 2). Six relevant performance categories selected by experts for performance assessment on a 7-point Likert scale.
**Learning Theory**	Mastery learning through deliberate practice guided by self-regulated learning.	Mastery learning through deliberate practice guided by self-regulated learning.
**Feedback Delivery**	Audiovisual metric-specific feedback provided autonomously and immediately depending on the participant's competency.	Live verbal debriefing with scripted feedback and instructions provided immediately depending on the participant's competency.
**Feedback Content**	Metric-specific videos played based on the learner's individual needs that describe the appropriate assessment criteria, demonstrate novice and expert performance examples, and provide actionable instructions to excel. Senior consultants with extensive subpial experience provided instructions and performance in the videos.	OSATS category-specific feedback prompts and actionable instructions used in a debriefing script that describes the relevant performance category and the lacking competency, and provides instructions tailored to the learner's individual needs. Feedback prompts and instructions were provided by senior consultants on how to excel.

© 2022 Fazlollahi AM et al. *JAMA Network Open.*

eFigure 6. Participant's Progression Through the VOA Training

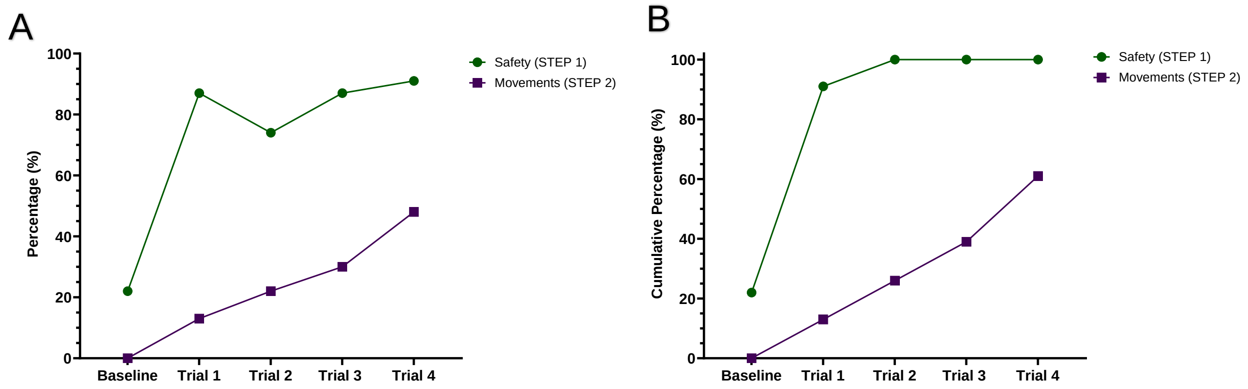

(A) Percentage of VOA participants who passed STEP-1 (safety) and STEP-2 (instrument movements) of VOA training at a specific trial. **(B)** Cumulative percentage of VOA participants who passed a specific VOA competency on or before a given trial. Data shows that the proportion of individuals who passed a competency at a given trial, were likely to pass that competency again in the following trial.

© 2022 Fazlollahi AM et al. *JAMA Network Open.*

eFigure 7. Instructor Group's OSATS Ratings During Practice

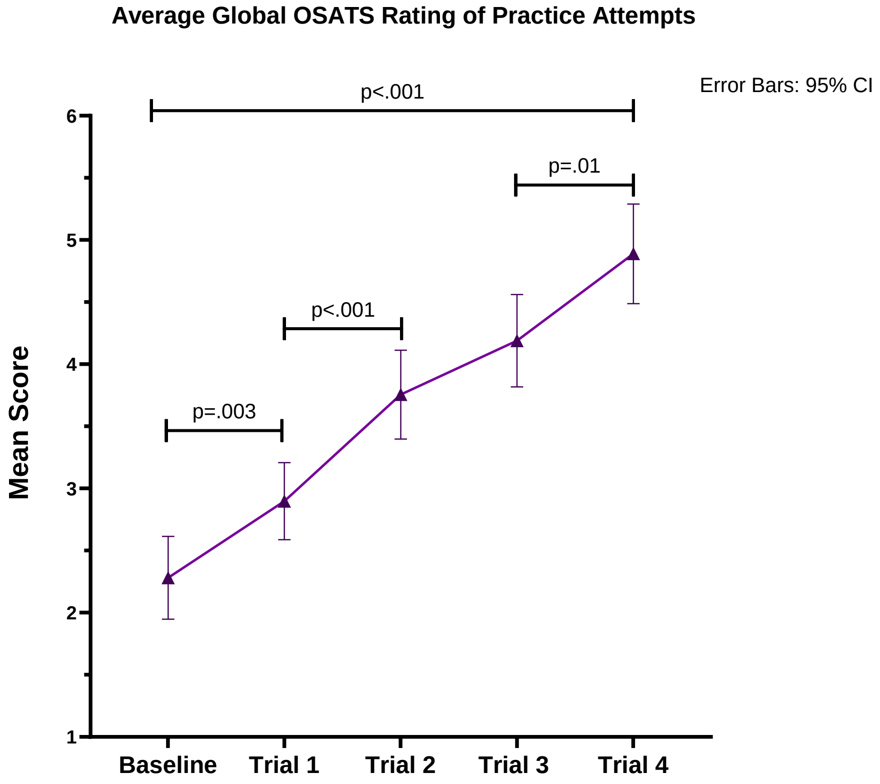

Average global OSATS ratings during practice scenario, measured as the mean of the 6 items in the visual rating scale. Instructor group's global OSATS ratings during practice shows performance improvement after debriefing and feedback sessions. In this group, average global OSATS scores improved by 0.62 points (95% CI 0.17-1.07, p=.003) from baseline at Trial-1, 0.86 points (95% CI 0.45-1.27, p<.001) from Trial-1 to Trial-2, 0.44 points (95% CI -0.09-0.95, p=.17) from Trial-2 to Trial-3, and 0.70 points (95% CI 0.14-1.26, p=.01) from Trial-3 to Trial-4. Figure above depicts the learning curve for participants in the instructor Group based on the OSATS scale. Bars represent the 95% confidence interval of the mean.

© 2022 Fazlollahi AM et al. *JAMA Network Open.*
