# Supplementary material for: Effect of Artificial Intelligence Tutoring vs Expert Instruction on Learning Simulated Surgical Skills Among Medical Students: A Randomized Clinical Trial
Source: JAMA Netw Open. 2022 Feb 22;5(2):e2149008. doi: 10.1001/jamanetworkopen.2021.49008 (PMC8864513; doi:10.1001/jamanetworkopen.2021.49008)
Supplement: Supplement 3. — Data Sharing Statement [file jamanetwopen-e2149008-s003.pdf]

Data Sharing Statement

Fazlollahi. Effect of Artificial Intelligence Tutoring vs Expert Instruction on Learning Simulated Surgical Skills Among Medical Students. *JAMA Netw Open*. Published February 22, 2022.
doi:10.1001/jamanetworkopen.2021.49008

Data
Data available: Yes
Data types: Deidentified participant data
How to access data: Upon request from authors Fazlollahi and Del Maestro. Contact ali.fazlollahi@mail.mcgill.ca and rolando.del_maestro@mcgill.ca.
When available: With publication

Supporting Documents
Document types: None

Additional Information
Who can access the data: Researchers whose proposed use of the data has been approved.
Types of analyses: Any purpose.
Mechanisms of data availability: With a signed data access agreement.
